# Preparation and Characterization of Doxycycline-Loaded Electrospun PLA/HAP Nanofibers as a Drug Delivery System

**DOI:** 10.3390/ma15062105

**Published:** 2022-03-12

**Authors:** Noémi-Izabella Farkas, Laura Marincaș, Réka Barabás, Liliana Bizo, Aranka Ilea, Graziella Liana Turdean, Monica Toșa, Oana Cadar, Lucian Barbu-Tudoran

**Affiliations:** 1 Department of Chemical Engineering, Faculty of Chemistry and Chemical Engineering, Babeș-Bolyai University, 11 Arany János Street, RO-400028 Cluj-Napoca, Romania; noemi.farkas@ubbcluj.ro (N.-I.F.); graziella.turdean@ubbcluj.ro (G.L.T.); 2 Department of Chemistry, Faculty of Chemistry and Chemical Engineering, Babeș-Bolyai University, 11 Arany János Street, RO-400028 Cluj-Napoca, Romania; laura.marincas@ubbcluj.ro; 3 Department of Chemistry and Chemical Engineering of Hungarian Line of Study, Faculty of Chemistry and Chemical Engineering, Babeș-Bolyai University, 11 Arany János Street, RO-400028 Cluj-Napoca, Romania; 4 Department of Oral Rehabilitation, Faculty of Dentistry, Iuliu Hațieganu University of Medicine and Pharmacy, RO-400012 Cluj-Napoca, Romania; aranka.ilea@umfcluj.ro; 5 Enzymology and Applied Biocatalysis Research Centre, Faculty of Chemistry and Chemical Engineering, Babeș-Bolyai University, 11 Arany János Street, RO-400028 Cluj-Napoca, Romania; monica.tosa@ubbcluj.ro; 6 INCDO-INOE 2000, Research Institute for Analytical Instrumentation, 67 Donath Street, RO-400293 Cluj-Napoca, Romania; oana.cadar@icia.ro; 7 Department of Molecular Biology and Biotechnology, Faculty of Biology and Geology, Babeş-Bolyai University, 1 Mihail Kogălniceanu Street, RO-400084 Cluj-Napoca, Romania; lucian.barbu@ubbcluj.ro; 8 National Institute for Research and Development of Isotopic and Molecular Technologies, 67-103 Donath Street, RO-400293 Cluj-Napoca, Romania

**Keywords:** electrospinning, polylactic acid, hydroxyapatite, doxycycline, drug delivery system, release kinetics

## Abstract

The present study aimed to prepare nanofibers by electrospinning in the system polylactic acid-hydroxyapatite-doxycycline (PLA-HAP-Doxy) to be used as a drug delivery vehicle. Two different routes were employed for the preparation of Doxy-containing nanofibers: Immobilization on the electrospun mat’s surface and encapsulation in the fiber structure. The nanofibers obtained by Doxy encapsulation were characterized using Fourier transform infrared (FTIR) spectroscopy, thermogravimetric (TG) and differential thermal analyses (DTA) and scanning electron microscopy (SEM). The adsorption properties of pure PLA and PLA-HAP nanofibers were investigated for solutions with different Doxy concentrations (3, 7 and 12 wt%). Moreover, the desorption properties of the active substance were tested in two different fluids, simulated body fluid (SBF) and phosphate buffer solution (PBS), to evidence the drug release properties. In vitro drug release studies were performed and different drug release kinetics were assessed to confirm the use of these nanofiber materials as efficient drug delivery vehicles. The obtained results indicate that the PLA-HAP-Doxy is a promising system for biomedical applications, the samples with 3 and 7 wt% of Doxy-loaded PLA-HAP nanofibers prepared by physical adsorption are the most acceptable membranes to provide prolonged release in PBS/SBF rather than an immediate release of Doxy.

## 1. Introduction

The electrospinning procedure is a frequently used technique in biomedicine [1], bone regeneration [2] and dental fields [3], with promising applications which include drug delivery carriers, tissue engineering and wound dressing [4]. The prevalence of this method is due to the excellent properties of the nanofibers. The electrospun nanofibers are considered to be promising candidates for biomedical applications due to their flexibility in imparting unique medical properties and high continuous production rates [5]. The nanofibers have a high specific surface area, variable porosity and biodegradability; their morphology is controllable [6]. Due to the properties mentioned above, nanofibers have increased the solubility and bioavailability of the drug. Consequently, the most studied area of nanofiber-based composites is in the design of drug delivery and controlled release systems. The method allows both the efficient binding of active anti-inflammatory drugs and antibiotics and the immobilization of enzymes and macromolecules.

The way of designing nanofiber-drug systems is influenced both by the nature and characteristics of the drug and its application purpose. Based on these, two methods are known: Immobilization on the electrospun mat’s surface and encapsulation in the fiber structure. The most important difference between the two methods is visible in the different drug releases [7]. Controlled release systems can be produced by several biopolymers and their mixtures, including polylactic acid (PLA), poly (lactide-co-glycolide) (PLGA), poly (ethylene-vinyl acetate) (PEVA) and polycaprolactone (PCL) [8]. The amount of released drug depends not only on the strength of the interactions between the polymer and the drug [9,10] but also on the diameter of the fibers [9] and the initial drug content used for electrospinning [11,12].

PLA has the appropriate biocompatibility, hydrophobicity and biodegradability [12]. The use of PLA as a biomaterial has been approved by the FDA (U.S. Food and Drug Administration) [13]. Compared to conventional drug dispensers [14], due to its better location specificity, PLA is used in bioresorbable sutures as vascular grafts and drug-eluting sutures, absorbable plates for fixation of fractures and drug delivery systems [13,15,16]. PLA scaffold with variable pore size allows efficient binding of different drugs and controlled drug release [17,18].

The addition of active molecules or biocompatible materials to electrospun fibers improves bioactivity and leads to solving various affinity problems. Nanostructures of CaP such as hydroxyapatite (HAP) are used to improve the bioactivity of electrospun polymer nanofibers since HAP has excellent biocompatibility and bioactivity. It was revealed that HAP-PLA composite nanofibers have high biocompatibility [19]. Similarly, due to the efficient nucleating effect, it was observed that HAP particles improve both the thermal and mechanical properties of PLA fibers [20]. The PLA/HAP composites produced by the electrospinning method are widely studied, mainly in bone repair studies. In recent years, it has been reported that the addition of HAP to polymeric nanofibers leads to a good in vivo tissue response [21,22]. The fiber membrane structure shows many similarities to the structure of the extracellular matrix, thereby facilitating not only the uptake and exchange of oxygen and nutrients [23] but also the drug release [24].

The encapsulation of antibiotics in PLA electrospun fibers plays a key role in the treatment of local wound and tissue reconstruction, as the method does not affect the bioactivity of the active substance [25]. For PLA/doxorubicin systems, drug release was studied for optimized and non-optimized fibers. In vitro release from optimized fibers in phosphate buffer is predominantly determined by the diffusion mechanism [26].

Doxycycline (Doxy) is a broad-spectrum antibiotic belonging to the tetracycline family. Doxy inhibits the production of protein by bacteria, which reduces their activity or leads to their complete death [27,28]. Due to the limited direct cell–drug contact, in the case of Doxy-loaded polymer-based systems, the dose-dependent cytotoxicity of the cells is eliminated [29]. Thus, the design and investigation of polymer-Doxy-based systems are primarily important in research. Subsequently adsorbed Doxy on poly (acrylic acid) nanofibers proved to be effective against gram-positive bacteria because the Doxy release followed a diffusion mechanism after the initial high release [30]. The same desorption mechanism was observed in the first hours of the Doxy-containing kefiran polymer-drug system [31]. Eskitoros-Togay et al. investigated the optimal composition of poly (ε-caprolactone)/poly (ethylene oxide) membranes in terms of Doxy release and activity. The results showed that a 3.5 wt% Doxy-containing membrane provides long-term drug release [32]. Moreover, for therapeutic application, various Doxy-containing PLA nanofibers have been investigated. In PBS, the dissolution of Doxy depends on the active substance content of the fibers; the increase in the amount of encapsulated Doxy also significantly increases the amount of Doxy released [12].

The electrospun fiber membranes have been a promising topic in the research area of drug delivery systems. PLA is widely used for electrospinning to fabricate electrospun nanofibers as drug delivery vehicles, whereas HAP and HAP-based composites have important applications in controlled drug delivery, drug conjugation and other biomedical treatments. Doxy is a tetracycline antibiotic used to manage and treat various bacterial infections. In addition, previous studies have demonstrated that fiber mats obtained by the electrospinning method may be suitable for the treatment of periodontal disease and they could provide a prolonged drug release [11].

Consequently, the aim of this study is based on common discussions between two research groups: Materials scientists and physicians. The novelty of this paper consists in the nanofiber’s composition and the perspective of a very concrete application, the treatment of periodontal disease. For the first time, to our knowledge, PLA-HAP-Doxy nanofibers were prepared by electrospinning. Two routes were employed for the preparation of Doxy-containing nanofibers—immobilization on the electrospun mat’s surface and encapsulation in the fiber structure—and the resulting materials were comparatively analyzed. Moreover, the desorption properties of the active substance were tested in two different fluids, SBF and PBS, to evidence the drug release properties.

## 2. Materials and Methods

### 2.1. Materials

The starting materials used for the preparation of PLA-based nanofiber systems were of an analytical degree, and their use was not preceded by any purification process. Polylactic acid (PLA, biopolymer, granule, 3 mm nominal granule size, weight 100 g, natural, Sigma Aldrich), dichloromethane (DCM, purity ≥ 99.5%, stabilized with 0.2% of ethanol, VWR Chemicals), chloroform (CHL, 99.0-99.4% (GC), Honeywell) and doxycycline hyclate (Doxy, purity ≥ 93.5%, Sigma Aldrich) were used for the preparation of PLA and PLA-based nanofibers. The starting materials for hydroxyapatite (HAP) synthesis were purchased from Carl Roth GmbH (Germany). The salts required for preparation of the simulated body fluid (SBF) and phosphate buffer solution (PBS) were obtained from Sigma Aldrich and “Reactivul” București.

### 2.2. Preparation of HAP, PLA and PLA-Based Nanofibers

For the HAP preparation, Ca(NO_3_)_2_·4H_2_O (purity ≥ 99%) and (NH_4_)_2_HPO_4_ (purity ≥ 98%) were used as precipitation reactants in aqueous solutions. In the reaction mixture, the theoretical Ca/P ratio of the aqueous solutions was 1.67; the pH 11 was adjusted with a 25% ammonia solution. After stirring the mixture for 24 h, the product was filtered and washed with distilled water. For further experiments, HAP was used as a precipitate.

PLA and PLA-based nanofibers were prepared by electrospinning (Fluidnatek^®^ LE-50 benchtop laboratory machine, Bioinicia S. L., Valencia, Spain). DCM/CHL mixture was used as a solvent in 6:4 vol%. For the pure PLA nanofiber, the solution contained 5 wt% polymers. For the production of PLA-HAP-Doxy, HAP precipitate and Doxy-loaded HAP with 3, 7 and 12 wt% were used. The fibers were spun (based on preliminary experiments) at ambient temperature, 25–27 kV voltage, 15 cm collector distance and a feeding rate of 1 mL/h. Detailed electrospinning conditions are shown in Table 1. The starting solutions and mixtures were dispensed from a 10 mL syringe, and the nanofibers were collected on baking paper, from which the electrospun mat could be easily removed.

### 2.3. Adsorption of Doxy on HAP Precipitate and PLA/PLA-HAP Nanofibers

Solutions with different concentrations (3, 7 and 12 wt%) of Doxy were used to prepare drug-loaded HAP materials. The appropriate amount of active substance was dissolved in distilled water and the adsorption process was carried out with constant stirring for 24 h at room temperature in a closed system. The phases were separated by centrifugation at 5500 rpm, 20 min. The concentration of Doxy in the liquid phase was examined spectrophotometrically at 274 nm. Doxy-loaded HAP samples were used to produce PLA nanofiber-based systems without drying.

The adsorption capacity of pure PLA and PLA-HAP nanofibers was also investigated for solutions containing 3, 7 and 12 wt% Doxy. For the measurements, 100 mg of the carrier and 5 mL of Doxy solution with appropriate concentration were used. The mixture was shaken for 24 h at 10 rpm using a Biosan Bio RS-24 equipment. After centrifugation, the liquid phase was examined spectrophotometrically at 274 nm. The solid material was dried to constant weight in a desiccator.

**Figure 1:**
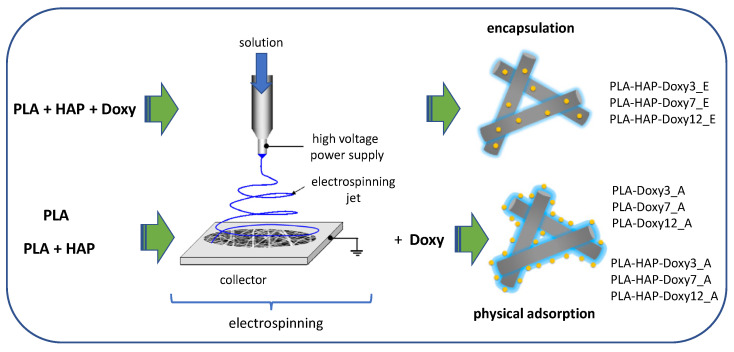
Schematic of two routes of drug loading and notation of the samples.

The scheme and notation of the samples obtained by Doxy encapsulation on the fiber structure and physical adsorption of Doxy on the electrospun mats are displayed in Figure 1.

### 2.4. Desorption Studies

The release mechanisms of drug-loaded HAP, PLA, PLA-HAP nanofibers and that of Doxy encapsulated PLA and PLA-HAP nanofibers were investigated spectrophotometrically at 274 nm. Desorption analyses were investigated in two salt solution, phosphate buffer (PBS) [33] and simulated body fluid (SBF) [34], at 37 °C.

In the drug release tests, 100 mg of PLA-based Doxy-containing samples were soaked in 5 mL of medium (PBS/ SBF) in sealed tubes and measured at defined time intervals (15, 30, 45, 60, 75, 90, 105, 120, 135, 150, 165, 180, 240, 300, 360, 420, 480, 720, 1440, 2880, 4320 and 5760 min). The experiments of drug release from the HAP precipitate were performed in 20 mL of PBS and SBF, respectively, with measurements at the same intervals.

For all measurements, 1 mL of sample was taken from the release medium and an equal volume of fresh PBS/SBF was added to maintain a constant volume.

For the corresponding cumulative percent of drug released, the points on the cumulative release curve represent the amount corresponding to time t, plus the sum of the amounts of active substance, corresponding to times t-n, i.e., those which have been removed and replaced with fresh buffer. The cumulative drug release (%) is the value calculated from the maximum drug content of the test sample and the amount of drug released at the measured t points.

### 2.5. Drug Release Studies

The mechanism of Doxy release for all Doxy-containing systems was investigated. To explain and understand the dissolution kinetics, fitting was performed for five commonly used models, including the zero-order kinetics, first-order kinetics, Higuchi model, Hixon–Crowell model and Korsmeyer–Peppas model. Linear regression analysis was performed on the whole dissolution interval (0–96 h), and the intervals were divided into 0–60 min, 60–360 min and 360–5760 min. The suitable model was selected based on the fit correlation coefficient R^2^.

### 2.6. Characterization Methods

The samples obtained by Doxy encapsulation in the fiber structure were analyzed by different methods. Fourier transform infrared (FTIR) spectra were collected using a Spectrum BX II (Perkin Elmer, Waltham, MA, USA) spectrometer on 1% KBr pellets measuring 12 mm in diameter, in the range of 4000–400 cm^−1^ using a resolution of 2 cm^−1^. Thermogravimetric (TG) and differential thermal analyses (DTA) of pure PLA and HAP-Doxy samples encapsulated in PLA fibers were performed with TA Instruments SDT Q600 equipment (New Castle, DE, USA) at a heating rate of 10 °C/min, up to 1000 °C under a nitrogen atmosphere. The morphology of PLA and encapsulated HAP-Doxy fibers was examined by scanning electron microscopy (SEM) using a CFEG SEM Hitachi SU8230 microscope (Japan). The success of drug binding on various carriers and the investigation of desorption of all Doxy-containing systems were measured with a Jasco V-650 UV-VIS double-beam spectrophotometer (Tokyo, Japan).

## 3. Results and Discussion

### 3.1. Fourier Transform Infrared Spectroscopy (FTIR) Analysis

**Figure 2:**
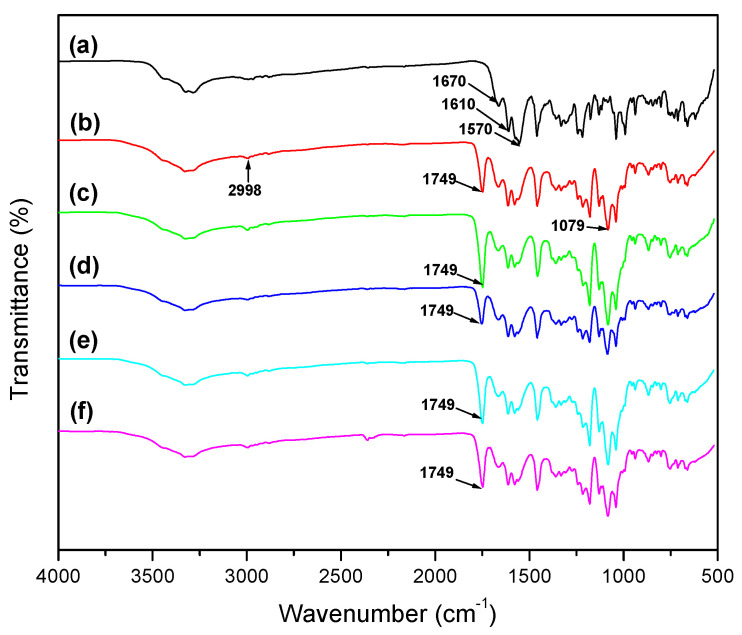
FTIR spectra of (**a**) Doxy, (**b**) PLA, (**c**) PLA-HAP, (**d**) PLA-HAP-Doxy3_E, (**e**) PLA-HAP-Doxy7_E and (**f**) PLA-HAP-Doxy12_E.

Fourier transforms infrared spectroscopy (FTIR) was used to analyze the interaction between different PLA-HAP and Doxy. The corresponding infrared spectra are presented in Figure 2.

The FTIR spectrum of Doxy shows characteristic bands between 3000 and 3500 cm^−1^ (νOH and νNH), and 1610 cm^−1^ (amide band I) and 1570 cm^−1^ (amide band II) [12,35,36]. Moreover, Doxy shows C=O and C=C stretches between 1700 and 1600 cm^−1^ [37]. The characteristic infrared bands of PLA show stretching frequencies for C=O, –CH_3_ asymmetric and C–O, at 1749, 2998 and 1079 cm^−1^, respectively. Moreover, bending frequencies for –CH_3_ asymmetric and –CH_3_ symmetric have been identified at 1452 and 1361 cm^−1^, respectively [37]. In the spectra of Doxy-loaded membranes, at about 1749 cm^−1^, the intensity of the main characteristic band of PLA increased when the amount of the Doxy in the membranes was increased too. When encapsulating 3, 7 and 12 wt% Doxy into the PLA-HAP nanofibers, characteristic bands in the range of 3000–3500 cm^−1^ and 1600–1500 cm^−1^ indicated the presence of Doxy in the PLA_HAP nanofibers. It could be implied that Doxy, HAP and PLA polymer were properly mixed [32].

### 3.2. Thermal Analysis

**Figure 3:**
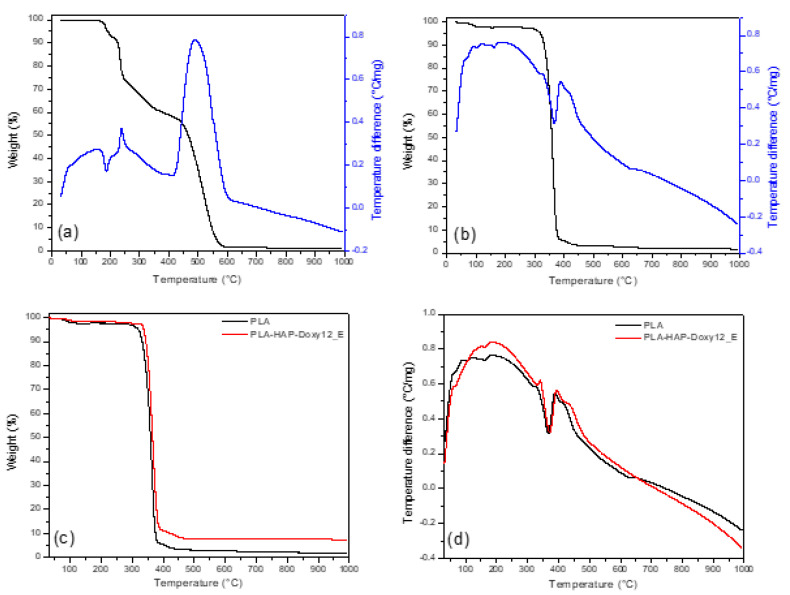
TG (black)/DTA (blue) plot of (**a**) Doxy and (**b**) PLA. Comparative (**c**) TG of PLA and PLA-HAP-Doxy12_E samples and (**d**) DTA curves of PLA and PLA-HAP-Doxy12_E samples (exo up).

One important aspect of the employment of thermal analysis when evaluating matrices for drug controlled-release is the fact that this method allows the investigation of the thermal stability of both the composite matrix and the drug, as well as the nature of dispersion of the drug in the composite matrix [38]. Figure 3 shows the thermal decomposition of Doxy, PLA, PLA_HAP and PLA-HAP-Doxy12_E electrospun nanofibers evaluated by TG/DTA analyses. Figure 3a shows the thermal decomposition of Doxy which begins its weight loss around 200 °C. PLA exhibits only one mass loss step due to the thermal decomposition of the PLA polymer chains around 385 °C (Figure 3b). The results also show that PLA follows a mechanism similar to the one reported by other authors, with only one stage of mass loss [39,40]. Figure 3c,d show the decomposition of PLA and encapsulated 12 wt% Doxy on PLA-HAP membrane. PLA-HAP_Doxy12_E composite nanofibers showed thermal resistance up to approximately 325 °C, after which sharp weight loss was observed. On the other hand, the thermal decomposition of PLA-HAP-Doxy12_E showed an additional increase in weight loss (~10%) compared to PLA. This difference in the thermal behavior of loaded and unloaded PLA-HAP with Doxy can be assigned to the successful adsorption of Doxy by PLA-HAP.

### 3.3. Scanning Electron Microscopy (SEM)

**Figure 4:**
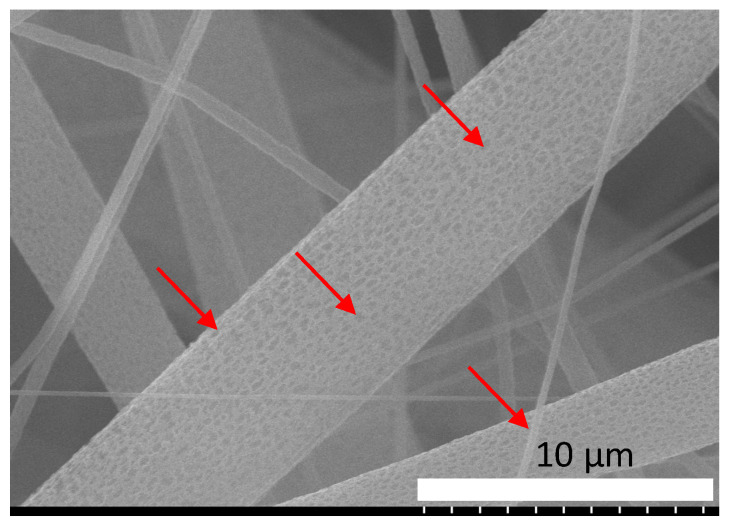
SEM images of PLA (8 wt%), electrospun membrane. The red arrows indicate the nanopores present on the surface of the fibers.

**Figure 5:**
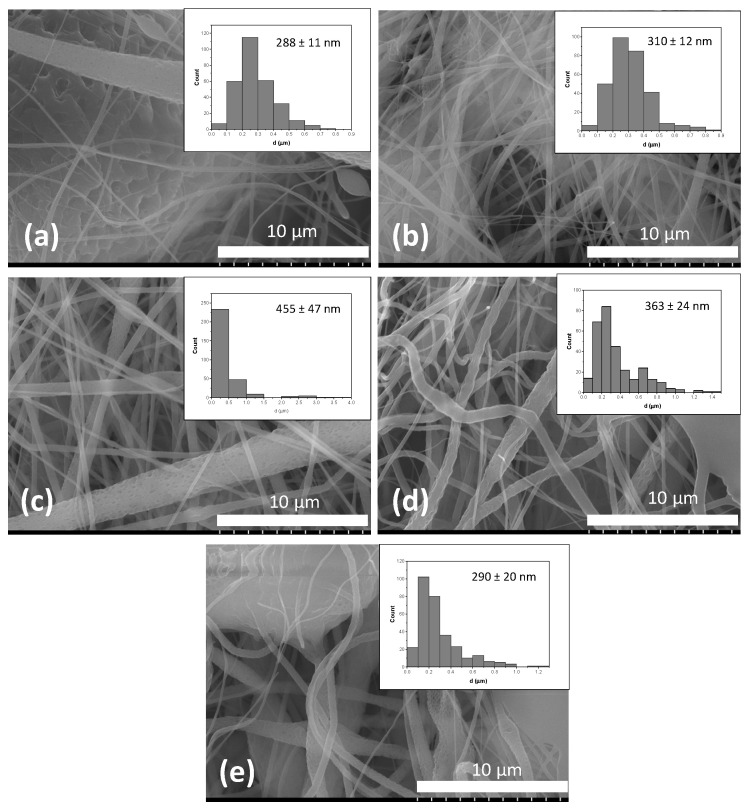
SEM images of (**a**) PLA (5 wt%), (**b**) PLA-HAP, (**c**) PLA-HAP-Doxy3_E, (**d**) PLA-HAP-Doxy7_E and (**e**) PLA-HAP-Doxy12_E electrospun membranes together with their corresponding histograms providing the fiber diameter distribution and average.

Figure 4 depicts the SEM image of electrospun PLA (8 wt%) nanofibers where the nanopores are indicated by red arrows. The PLA (5 wt%), PLA-HAP and PLA-HAP-Doxy nanofiber membranes images are revealed in the Figure 5. The images show randomly oriented fibers with nanopores clearly observed on the surface of the nanofibers.

The formation mechanism of the porous structure of the fibers can be attributed to the phase separation process of the polymer-solvent, which occurs during the electrospinning process, as previously reported by Doan et al. and Liang et al. [41,42]. The authors showed that the morphology and the interior structure of the fibers can be controlled through the adjustment of the solvent ratio. This type of structure was found to enhance the functionality of nanomaterials and improve pore connectivity and surface areas of electrospun nanofibers [43].

The membranes have a narrow fiber diameter distribution. The average fiber diameter was calculated from Figure 5 and displayed on the inset. For the calculation, the diameters of the fibers were measured by ImageJ software and the data analysis (statistical analysis) was performed in Origin. The average fiber diameter was expressed as mean ± standard deviation. The average fiber diameter ranges from 215 to 478 nm. As shown in Figure 5, while pure PLA had 288 ± 11 nm average fiber diameter, the PLA-HAP membrane exhibited an increase in average fiber diameter to 310 ± 12 nm. Furthermore, a significant increase in the average fiber diameters was observed for PLA-HAP-Doxy membranes. However, membranes with the highest Doxy amount exhibited the lowest average fiber diameter to 290 ± 20 nm. Increasing the amount of Doxy on the samples significantly decreased average fiber diameter. In our study, the average fiber diameter of PLA-HAP-Doxy membrane loaded with 3 wt% Doxy was higher than those of the other membranes with higher content of Doxy. This phenomenon was also observed in the case of PLGA- poly(D,L-lactide-co-glycolide) nanofibers and doxycycline-embedded nanofibers, where the fiber diameter decreased significantly upon the addition of doxycycline [44].

### 3.4. Adsorption Study

#### 3.4.1. Adsorption Study on HAP Precipitate

**Figure 6:**
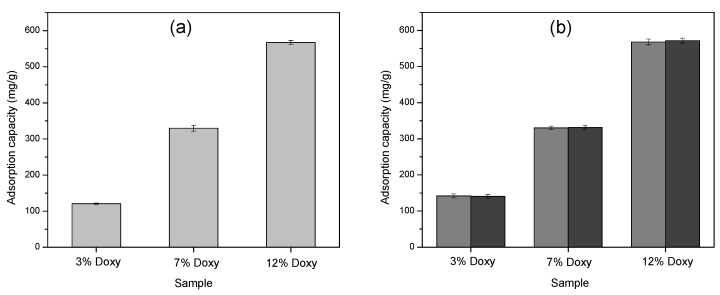
The adsorption capacity of different concentrations of Doxy loaded onto (**a**) HAP precipitate (light grey), and (**b**) PLA (grey)/PLA-HAP (dark grey) nanofibers, respectively. The error bars represent the standard deviation of 3 replicates.

Figure 6a shows the average adsorption capacities of HAP in the case of Doxy solutions with different initial concentrations. Individual measurements showed standard deviation of less than 6% in all cases. Physical adsorption of Doxy on the HAP precipitate was successful, with an average adsorption efficiency of 82-95%. The adsorption capacity increased when the concentration of the initial Doxy solutions increased; the adsorption capacity of HAP was 120.86 mg/g and 576.3 mg/g for (in the case of) 3 g/L and 12 g/L Doxy, respectively.

The adsorption process was mostly influenced by the concentration of the Doxy solutions [26]. The high Doxy content of HAP was probably due to the intensification of interactions between Doxy-HAP functional groups with increasing initial Doxy concentration [45]. The formation of interactions was enhanced by the possibility of collisions between the drug molecules and the HAP surface, and the mass transfer from solution towards the solid phase increased. Doxy-containing HAP samples were subsequently encapsulated in PLA nanofibers by the electrospinning method, and the drug release was also investigated.

#### 3.4.2. Adsorption Study on PLA Based Nanofibers

The simplest way to design drug-containing nanofiber-based drug delivery systems is through physical adsorption [46]. The success of the physical adsorption on the PLA and PLA-HAP nanofibers studied in Doxy-containing solutions with different initial concentrations is illustrated in Figure 6b. The change in the adsorption capacity is similar to the one discussed for HAP, increasing in value with increasing concentration of Doxy solution.

HAP encapsulated in PLA nanofiber did not significantly affect the adsorption capacity of the PLA nanofiber, the final Doxy content being similar for PLA and PLA-HAP samples. Consequently, the formation of secondary interactions between the functional groups of the drug and the carriers, and the achievement of high adsorption capacity were facilitated by the network-like structure and spatial arrangement of the fibers [47], as evidenced in Figure 6b.

### 3.5. Investigation of Drug Release

**Figure 7:**
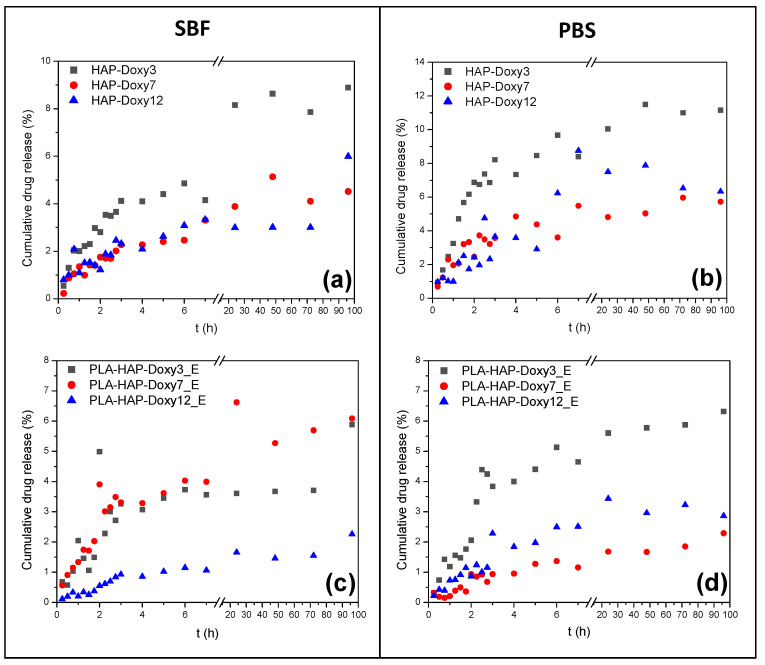
The cumulative release profile of encapsulated Doxy from (**a**,**b**) HAP and (**c**,**d**) PLA-HAP nanofibers in (**left**) SBF and (**right**) PBS, respectively.

The drug release from the Doxy-loaded HAP precipitate was compared with the drug release from HAP-Doxy carriers encapsulated in PLA nanofibers. The initial concentration of Doxy was the same on the HAP precipitate carrier as in the PLA_HAP fibers. Figure 7 illustrates the drug release behavior of the six samples soaked in SBF (Figure 7a,c) and PBS (Figure 7b,d), respectively.

Doxy dissolution from HAP showed a sustained trend in SBF (Figure 7a); after 96 h of measurement, the amount of the released drug did not exceed 9% in any of the samples. In the first six hours, the samples HAP-Doxy7 and HAP-Doxy12 showed very similar release profiles. The amounts of dissolved active substance in these cases were 2.46% and 3.07%, while the HAP-Doxy3 sample showed a faster release, 4.86%. The burst release from the HAP-Doxy3 was even more evident in PBS (Figure 7b) because in this case 86% of the amount of the Doxy dissolved in 96 h was released in the first six hours. Samples HAP-Doxy7 and HAP-Doxy12 showed similar and irregular dissolution profiles in PBS. The dissolution of the Doxy was higher in PBS than in SBF in all three samples, but this was not significantly different for samples with high Doxy content.

The release profiles of the HAP-Doxy samples encapsulated in the PLA fibers are shown in Figure 7c,d. The drug dissolution showed a sustained release both in SBF and PBS because the release was inhibited due to the encapsulation of the drug-loaded HAP in the polymer fibers. The dissolution profiles were different from those discussed for HAP. It could be observed, even in this case, that the desorption depended on the investigated medium. In SBF, during the first six hours, the dissolution of Doxy from the PLA-HAP-Doxy3_E and PLA-HAP-Doxy7_E samples was very similar; then the PLA-HAP-Doxy7_E sample showed an increasing dissolution tendency, while the release from the PLA-HAP-Doxy3_E sample was in equilibrium for days. After 96 h, similar amounts of Doxy were released from the two samples (5.88% and 6.08%, respectively). At high Doxy content, the drug release profile showed the slowest dissolution over the time of measurement: Only 2.25% of the Doxy was released.

While the amount of the drug dissolved was smaller for sample PLA-HAP-Doxy3_E than for HAP-Doxy3, the dissolution profile was similar. The PLA-HAP-Doxy3_E composite showed a continuously increasing dissolution in PBS. Similarly, it was observed that the higher the Doxy content of the sample, the lower amount of drug was detected in the SBF/PBS solutions. Since the release of the drug from the fibers is a rather complex process, it is difficult to explain our observations. It is conceivable that in the case of a sample with high Doxy content, the large amount of active substance results in a decrease in the rate of release [48] and, conversely, a small amount of active substance may lead to an accelerated release [49]. Yamawaki-Ogata et al. experimented the in vitro release of Doxy from biodegradable PLA mixed with Doxy prepared by electrospinning technique. The results of the release performed in PBS revealed that approximately only 7% of the Doxy was released into the solution from the fibers on the first day [50].

**Figure 8:**
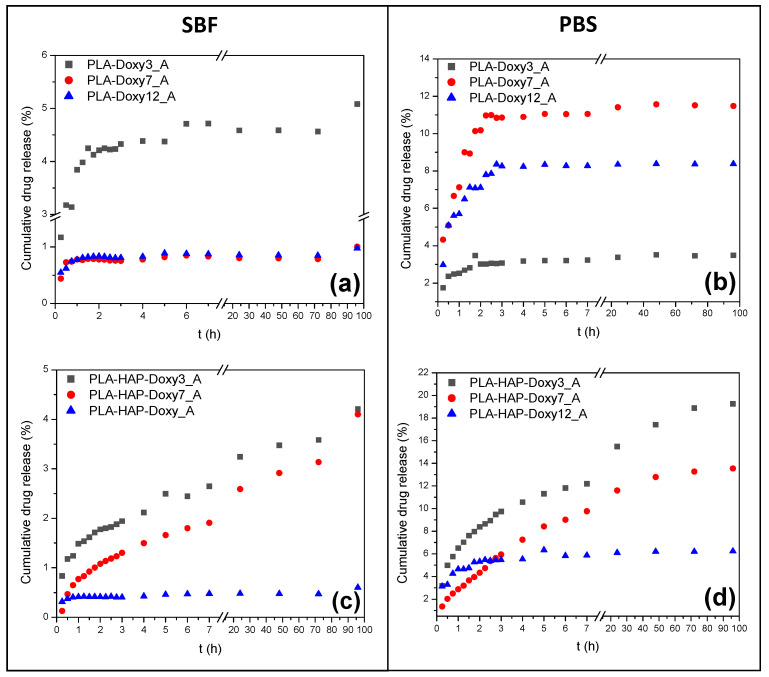
The cumulative release profile of physically adsorbed Doxy from (**a**,**b**) PLA and (**c**,**d**) PLA-HAP nanofibers in (**left**) SBF and (**right**) PBS, respectively.

The release of Doxy physically adsorbed on PLA and PLA-HAP fibers was examined in SBF and PBS (Figure 8). The release from Doxy-loaded PLA nanofibers showed a similar profile in SBF in all three cases. Burst release from the nanofibers could be observed in the case of the least Doxy amount; 92.7% of the total desorbed amount was already released within the first six hours. There was no significant difference between the release profiles of the PLA-Doxy7_A and PLA-Doxy12_A samples; in both cases an increased dissolution period after the first hour was observed.

The systems discussed previously showed different behaviors in PBS, the amount of drug released was reversed compared to the amount in SBF. Depending on the Doxy content, the dissolution profiles were clearly separated. The cumulative release was nearly linear; it showed abruptly increasing behaviors in the first four hours. This trend was significant for samples PLA-Doxy7_A and PLA-Doxy12_A, where the amount of drug released was 10.9% and 8.23%, respectively (the value was less than 1% in SBF for both systems). Further examination of the systems revealed that the amount of Doxy dissolved was almost constant; all three samples reached the equilibrium state. The drug dissolution was regular; the cumulative Doxy content in PBS did not change over time.

It could be concluded that the presence of HAP in the PLA fibers affected the dissolution profiles of Doxy. The PLA-HAP-Doxy3_A and PLA-HAP-Doxy7_A systems showed an obvious increasing dissolution trend in both SBF and PBS. The amount of Doxy released from these samples in PBS was significant, more than four and three times more drugs being dissolved over the measured time. Due to its amino, carboxyl and hydroxyl functional groups, Doxy is a highly polar compound. This property results in a rapid release in PBS [32].

Less than 1% of the drug was dissolved from the PLA-HAP-Doxy12_A sample in SBF, so in this case, the presence of HAP had no significant effect on the process. The dissolution curve was similar in PBS, but higher concentrations were also detected in the investigated medium. Both cases reached equilibrium quickly.

During the adsorption to fiber-based systems, the adsorption accrued not only at the surface but also in the space between the fibers. The active substance bound to the surface led to a fast release, while the release of drug from the porous parts of the network slowed [51]. Consequently, at low concentrations, the drug was desorbed from the surface, resulting in a fast process and a higher amount of dissolved drug, while at high Doxy content, dissolution occured not only from the surface but also from the inside of the mats. The diffusion of the investigated medium into the sample was also an important influencing factor, which in our case resulted in less drug release.

Mathematical models describing drug release were used to explain the release profiles and to investigate the release mechanism.

### 3.6. Drug Release Studies

Detailed investigations for a better understanding of the release of Doxy from PLA-based nanofiber systems were performed, using kinetic mathematical models. Regression analyses were carried out for drug dissolution in both SBF and PBS solutions, for all samples.

The regression coefficients (R^2^) obtained for Doxy-loaded HAP samples encapsulated in PLA fibers and Doxy-loaded PLA fibers prepared by physical adsorption are shown in Table 2 and Table 3, respectively. The drug release from nanofibers was also a complex process, and the nature of the drug, polymer, additives and solvent also indirectly affected the drug release [52]. Based on these, the fitting to the models was performed not only for the total observation period but also for subintervals. Since no acceptable fittings have been found in these cases, Table 2 and Table 3 contain only the correlation coefficient values found for the total time interval. The R^2^ values in the tables show that the Doxy dissolution cannot be explained by any of the studied models, for any of the samples, regardless of the medium in which the studies have been performed.

Table 4 shows the regression analysis of Doxy release (in SBF) from Doxy-containing PLA-HAP nanofibers prepared by physical adsorption. In this case, significantly better results were found. While for the PLA-HAP-Doxy3_A and PLA-HAP-Doxy7_A samples, the fittings followed the release according to the Korsmeyer–Peppas model, the division into intervals allowed for an even more detailed discussion [53]. The model provided an answer to whether the dissolution follows Fickian diffusion or not. In solving this question, the value of “n”, i.e., the slope of the fit, provided an answer [54]. In our case, the dissolution in the first hour was regulated by n < 0.5, so clearly by Fickian diffusion. The release within 1–96 h for the PLA-HAP-Doxy12_A sample did not show an adequate fit for either model, and the PLA-HAP-Doxy3_A and PLA-HAP-Doxy7_A samples followed the Higuchi model.

Regression analysis demonstrated that drug dissolution from the PLA-HAP-Doxy3_A and PLA-HAP-Doxy7_A samples was controlled by diffusion. The appropriate fit to the Korsmeyer–Peppas and Higuchi models confirmed a prolonged release, which was also observed in the desorption profiles [55].

Fittings with kinetic models were also performed for release in PBS. The correlation coefficient values are summarized in Table 5. Based on these values, the dissolution mechanism of Doxy also proved to be the best for the Korsmeyer–Peppas model (see bold values from Table 5). PLA-HAP-Doxy12_A did not show modelable behavior in PBS. From the PLA-HAP-Doxy3_A and PLA-HAP-Doxy7_A samples, the Doxy dissolution was mainly by Fickian diffusion, the value of “n“ was less than 0.5 in most cases. It is important to note that, in the first 6 h, the swelling of the polymer fibers was also a driving force for drug release, as “n“ reached a value between 0.5 and 1 [31].

## 4. Conclusions

In the present study, a novel membrane was developed from hydrophobic PLA polymer and biocompatible HAP, as a potential drug delivery vehicle for the prolonged release of Doxy. Two routes were employed for the preparation of Doxy-containing nanofibers: Immobilization on the electrospun mat’s surface and encapsulation in the fiber structure. Moreover, mathematical models describing drug release were used to explain the release profiles and to investigate the release mechanism for both Doxy-loaded HAP samples encapsulated in PLA fibers and Doxy-loaded PLA/HAP fibers prepared by physical adsorption. The drug release from nanofibers, either in SBF or PBS, was a complex process influenced by the nature of the drug, polymer, additives and solvent, which indirectly affected the drug release. Consequently, the Doxy dissolution could not be explained by any of the studied models. Conversely, significantly better results were found in the case of Doxy-containing PLA/HAP nanofibers prepared by physical adsorption. Regression analysis demonstrated that the drug dissolution in SBF/PBS, from samples with 3 and 7 wt% Doxy, was controlled by diffusion. The appropriate fit to the Korsmeyer–Peppas and/or Higuchi models confirmed a prolonged release, which was in agreement with the desorption profiles.

The results have shown that the Doxy-containing PLA-HAP nanofibers prepared by physical adsorption with low Doxy content (3 and 7 wt%) are the most acceptable membrane due to providing a prolonged release rather than an immediate release of the drug. In conclusion, this novel material may be a good candidate as a drug delivery vehicle for the drug delivery system.

## Figures and Tables

**Table 1:** The electrospinning conditions for the production of membranes and average fiber diameter.

Sample ID	Electrospinning Conditions			
	PLA/HAP Ratio	Collector Distance (mm)	Flow Rate (mL/h)	Voltage (kV)
PLA	1/0	150	1	25–27
PLA-HAP	0.8	150	1	27
PLA-HAP-Doxy3_E	0.8	150	1	27
PLA-HAP-Doxy7_E	0.8	150	1	27
PLA-HAP-Doxy12_E	0.8	150	1	27

**Table 2:** Release kinetic models and regression coefficients (R^2^) of encapsulated Doxy on PLA-HAP nanofibers, in SBF and PBS (0–96 h).

Samples	Medium	Zero-Order	First Order	Higuchi	Hixson-Crowell	Korsmeyer-Peppas
PLA-Hap-Doxy3_E	SBF	0.3518	0.3556	0.4584	0.3544	0.6307
	PBS	0.3407	0.3443	0.2292	0.3431	0.8003
PLA-HAP-Doxy7_E	SBF	0.4648	0.4697	0.662	0.4681	0.8888
	PBS	0.5661	0.5687	0.7699	0.5678	0.8907
PLA-HAP-Doxy12_E	SBF	0.6163	0.6185	0.7764	0.6177	0.9012
	PBS	0.3637	0.3648	0.1549	0.3644	0.8224

**Table 3:** Release kinetic models and regression coefficients (R^2^) of physically adsorbed Doxy on PLA nanofibers, in SBF and PBS (0–96 h).

Samples	Medium	Zero-Order	First Order	Higuchi	Hixson-Crowell	Korsmeyer-Peppas
PLA-Doxy3_A	SBF	0.1578	0.1604	0.2683	0.1595	0.4139
	PBS	0.2522	0.2536	0.4039	0.2531	0.6493
PLA-Doxy7_A	SBF	0.2455	0.2461	0.2984	0.2459	0.4004
	PBS	0.147	0.15	0.2807	0.149	0.5519
PLA-Doxy12_A	SBF	0.2218	0.2221	0.3257	0.222	0.5499
	PBS	0.1289	0.1306	0.2585	0.13	0.5212

**Table 4:** Release kinetic models of physically adsorbed Doxy on PLA-HAP nanofibers, in SBF.

Kinetic Models	Time (h)	Samples		
		PLA-HAP-Doxy3_A	PLA-HAP-Doxy7_A	PLA-HAP-Doxy12_A
		Values of R^2^		
Zero-order	0–96	0.6875	0.777	0.5753
First order	0–96	0.6915	0.7815	0.5756
Hixson–Crowell	0–96	0.6902	0.78	0.5755
Higuchi	0–1	0.9573	0.9833	0.9449
	1–6	0.9676	0.9989	0.5755
	6–96	0.942	0.948	0.444
	0–96	0.8589	0.9137	0.6665
Korsmeyer- Peppas	0–1	0.9588	0.9992	0.981
	1–6	0.935	0.9838	0.461
	6–96	0.9378	0.9073	0.3444
	0–96	0.9797	0.9677	0.7706

**Table 5:** Release kinetic models of physically adsorbed Doxy on PLA-HAP nanofibers, in PBS.

Kinetic Models	Time (h)	Samples		
		PLA-HAP-Doxy3_A	PLA-HAP-Doxy7_A	PLA-HAP-Doxy12_A
		Values of R^2^		
Zero-order	0–96	0.6817	0.5498	0.2373
First order	0–96	0.7044	0.5632	0.2396
Hixson–Crowell	0–96	0.6969	0.5587	0.2388
Higuchi	0–96	0.8627	0.7554	0.4028
Korsmeyer- Peppas	0–1	0.9817	0.9981	0.8498
	1–6	0.9955	0.9953	0.8415
	6–96	0.9944	0.9467	0.9719
	0–96	0.9382	0.8858	0.6772

## Data Availability

Not applicable.
